# An Epilepsy Detection Method Using Multiview Clustering Algorithm and Deep Features

**DOI:** 10.1155/2020/5128729

**Published:** 2020-08-01

**Authors:** Qianyi Zhan, Wei Hu

**Affiliations:** ^1^School of Artificial Intelligence and Computer Science, Jiangnan University, Wuxi, China; ^2^Jiangsu Key Laboratory of Media Design Software Technology, Wuxi, China; ^3^Department of Nuclear Medicine, Nanjing Medical University, Affiliated Wuxi People's Hospital, Wuxi, China

## Abstract

The automatic detection of epilepsy is essentially the classification of EEG signals of seizures and nonseizures, and its purpose is to distinguish the different characteristics of seizure brain electrical signals and normal brain electrical signals. In order to improve the effect of automatic detection, this study proposes a new classification method based on unsupervised multiview clustering results. In addition, considering the high-dimensional characteristics of the original data samples, a deep convolutional neural network (DCNN) is introduced to extract the sample features to obtain deep features. The deep feature reduces the sample dimension and increases the sample separability. The main steps of our proposed novel EEG detection method contain the following three steps: first, a multiview FCM clustering algorithm is introduced, and the training samples are used to train the center and weight of each view. Then, the class center and weight of each view obtained by training are used to calculate the view-weighted membership value of the new prediction sample. Finally, the classification label of the new prediction sample is obtained. Experimental results show that the proposed method can effectively detect seizures.

## 1. Introduction

Epilepsy is a common mental disease in neurology. It is a chronic neurological disease caused by sudden and temporary disturbance of brain function due to the paroxysmal abnormal discharge of brain neurons [1]. Worldwide, more than 1% of the population suffers from epilepsy [2]. Among neurological diseases in China, epilepsy has become the second most common disease, with fewer patients than headaches. There are many clinical manifestations of seizures. The more common symptoms are temporary sensory disturbances, behavioral disturbances, limb convulsions, loss of consciousness, or abnormal neurological function. Epilepsy has caused serious harm to patients' health and in some cases has been even life-threatening [3]. It can be said that epilepsy has brought a great burden to human health and social development. Therefore, the study of epilepsy disease has very important significance for medical research and the development of human society.

At present, the diagnosis of epilepsy is mainly through the patient and his relatives and friends, as much as possible to obtain a complete and detailed history of the patient's seizure and then be referred to the EEG for examination. The determination of seizures is mainly based on the patient's clinical manifestations and EEG records. EEG can record the abnormal electrical activity of neurons in patients' brains during epileptic seizures, so it has become an important tool for exploring the characteristics of epilepsy. It has been widely used in various epilepsy studies including epilepsy diagnosis, seizure location, qualitative research, seizure prediction, and seizure control. Until now, electroencephalogram examination is still mainly carried out by medical staff visual observation combined with their own experience. However, relying on manual recognition of epilepsy EEG has many drawbacks. First, with the advancement of medical conditions and the development of EEG acquisition equipment, the amount of EEG data is getting larger and larger, and the duration of EEG of a patient generally exceeds tens or even hundreds of hours. In the face of a large amount of EEG data, manual detection is time-consuming and efficient. Second, some features of epilepsy on EEG are very subtle and difficult to recognize by human eyes alone. Third, when medical staffs work for a long time, their judgment will be affected, which will easily lead to an increase in the false detection rate. With the in-depth research and development of computer technology, automatic detection and recognition of epilepsy EEG signals using computers as auxiliary tools have become an important auxiliary detection method. The study of automatic epilepsy detection technology becomes extremely important.

In view of the fact that EEG signals are a type of time series, the time-frequency analysis method is widely used when researching automatic epilepsy detection algorithms. Such methods analyze the time domain or frequency domain of epilepsy EEG signals or combine the two to capture the characteristic information of epilepsy EEG signals. Some time-frequency analysis methods such as wavelet analysis [4, 5], S transform [6], and empirical mode decomposition [7, 8] are used to analyze the characteristics of epilepsy EEG signals. Due to the nonlinear characteristics of EEG signals, nonlinear dynamic analysis has also become a method in the automatic detection of epilepsy. Entropy, as a parameter representing the complexity and randomness of the signal, is used as a feature of epilepsy EEG signals to distinguish it from EEG signals of different periods [9, 10]. With the development of pattern recognition and machine learning research, various learning algorithms [11–20] are also widely used in automatic detection of epilepsy. In recent years, with the development of deep learning, some deep neural networks have also been used in the classification of epilepsy EEG signals [21, 22].

At present, epilepsy detection technology has made great progress and many important research results have been achieved, but there are also some problems in the existing research. For example, (1) in the study of epilepsy detection based on EEG signals, different features such as the time domain and time-frequency domain of EEG are often directly combined with machine learning algorithms to identify different EEG patterns [23–26]. Different EEG feature extraction methods and classification methods are time-consuming and laborious and cannot meet real-time requirements in actual production. (2) Use one of EEG's features for decision-making, ignoring data from other perspectives, and making the decision result less than ideal. (3) The traditional feature extraction method applied to the features obtained by multiview data is easy to lose some important information and is not suitable for the feature extraction of multi-view data. (4) In terms of classifier design, the classifier based on a single perspective does not consider the correlation between data, and the decision result is not comprehensive enough. (5) When many existing epilepsy detection systems are applied to actual clinical data, it is difficult to achieve the performance results obtained on the experimental data set; that is, the robustness of the detection system is relatively low. Based on the above research status and analysis of existing problems, this paper proposes an epilepsy detection method using multiview clustering algorithm and deep features. The specific contributions of this study are summarized as follows:

1. Construct the original multiview data set of epilepsy EEG. Different EEG analysis methods were used to obtain the data set under three perspectives of spectrum signal, time domain signal, and time domain signal of EEG
2. Extract deep features. Use deep DCNN to extract deep features of EEG signals from 3 perspectives, respectively, so as to obtain deep features from each perspective. This deep feature has a better ability to recognize epilepsy. And construct a multiview data set with deep features
3. Train a multiview classifier. First, a multiview FCM clustering algorithm is introduced, and the training samples are used to train the center and weight of each view. Then, the class center and weight of each view obtained by training are used to calculate the view weighted membership value of the new prediction sample. Finally, the classification label of the sample is obtained

## 2. Related Works

### 2.1. Epilepsy Detection Process

**Figure 1:**
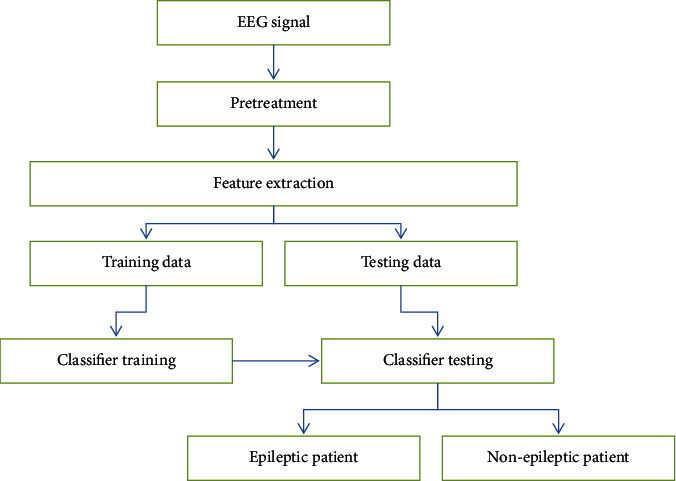
Flow chart of epilepsy detection.

The EEG signals of patients with epilepsy are generally divided into four periods, namely, interseizure period, preseizure period, seizure period, and late seizure period. The interval between seizures refers to the EEG signals collected by epilepsy patients when the seizures are not occurring. The preseizure period refers to the EEG signals that may be collected when epilepsy is about to occur. There is no clear regulation on the length of this period and the starting position. The seizure period refers to the EEG signals recorded during the patient's seizure. Late seizures generally refer to the collection of EEG signals when the brain is in a recovered state after the seizure has ended. The process of predicting seizures can generally be divided into the following steps. The first step is to preprocess the EEG signals. In the second step, feature extraction is performed on the preprocessed samples to reduce the sample dimensions. The third step is to use the training samples to train the classifier. The fourth step is to use the trained classifier to classify the test samples to identify whether they are epilepsy patients. The detection flow chart is shown in Figure 1.

### 2.2. EEG Signal and Its Characteristics

As an important tool for researchers to study brain function, EEG reflects the electrical activity of brain nerve cell groups on the surface of the cerebral cortex or scalp. EEG is obtained by recording continuous, spontaneous, rhythmic potential changes in the brain nerve cell population through electrodes placed in the cortex or scalp. Certain characteristics of EEG signals are usually used as an important basis for the diagnosis of brain diseases. Because it contains a large amount of physiological and pathological information, it has played a huge role in clinical medicine, especially for the location and characterization of epilepsy.

The current mainstream EEG analysis methods are the time domain analysis method [27–29], frequency domain analysis method [30–33], time frequency domain analysis method [34, 35], and nonlinear dynamics method [36–39]. The time-domain analysis method mainly analyzes the time-domain waveforms of EEG signals and extracts waveform features such as period and rhythm as the basis for detecting seizures. Its intuitiveness is strong, and its physical meaning is clear. Frequency domain analysis of EEG signals is mainly based on Fourier transform or power spectrum estimation. Analyze the power spectrum distribution of EEG signals and the components of EEG signals in each frequency band. Because EEG signals are random and nonstationary, simple time-domain analysis and frequency-domain analysis cannot provide the joint distribution information of EEG signals in the time domain and frequency domain and cannot describe the change of signal frequency with time. Therefore, a time-frequency analysis method suitable for nonstationary signal processing is used for epilepsy EEG analysis and feature extraction. The EEG data obtained by different analysis methods have different characteristics. Common EEG data features are shown in Table 1.

## 3. Deep Convolutional Neural Network

In the 1960s, when Hubel and Wiesel studied the cat's cerebral cortex, they discovered that the cat's neurons for direction selection and local sensitivity formed a unique network structure. This structure greatly reduces the complexity of the artificial neural network and the number of parameters [40]. DCNN borrows from this network structure to form a unique feedforward neural network. In 1998, Lecun et al. proposed the LeNet-5 network model [41], but after a long period of time, the development of CNN fell into stagnation. In 2006, Hinton and Salakhutdinov pointed out that the feature learning ability of neural networks with multiple hidden layers is more powerful [42]. Since then, the structure of DCNN has been continuously improved by researchers [43], and models such as VGG [44], Google net [45], and Res net [46] have appeared successively. Compared with general neural networks, DCNN has four important characteristics: local connections, shared weights, pooling layers, and deeper network structures [47]. It is these characteristics that make the DCNN have more advantages than the general neural network when processing multiple types of signals.

**Figure 2:**
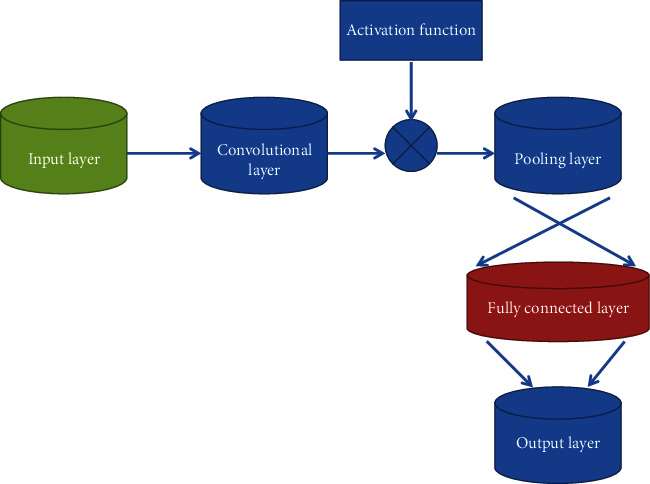
DCNN structure.

In this paper, the DCNN model is used to model the EEG data of patients with epilepsy to achieve automatic learning of epilepsy features. The DCNN structure used in the experiment is shown in Figure 2. The DCNN model is mainly composed of convolutional layers and pooling layers, and ReLu and Dropout layers can be inserted between them to improve network performance.

### 3.1. Multiview Clustering

Current data sets often have high dimensionality and complexity. Complex data sets usually contain many different and reasonable clustering patterns. For example, in document data classification, there are both topic-based models and writing styles. In movie classification, it can be classified according to the type of movie or according to the genre of the movie. In gene classification, clustering can be based on the structure and function of genes.

**Figure 3:**
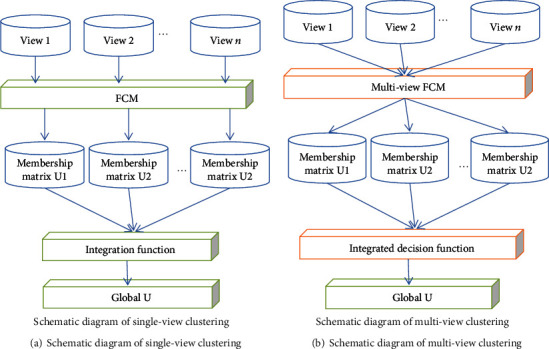
Comparison of single-view and multiview clustering algorithm schematics.

When traditional clustering algorithms analyze data, they usually only focus on a single clustering pattern in the data. To understand data from a single perspective, we cannot have a comprehensive analysis and understanding of complex data. This problem has gradually attracted the attention of scholars and promoted the rapid development of multiview clustering. The multiview clustering algorithm hopes to mine multiple different and reasonable clustering structures from the data. There are two basic requirements. (1) The quality of the clustering result is high; that is, it can capture some reasonable internal structure contained in the data set itself. (2) There is no redundancy between different clustering results. Analyze the data from multiple perspectives, and discover multiple different clustering patterns in the data. The comparison between the traditional single-view clustering and multiview clustering schematics is shown in Figure 3 (using the classic FCM algorithm as an example).

## 4. Introduction of the Proposed Algorithm

### 4.1. Algorithm Framework

**Figure 4:**
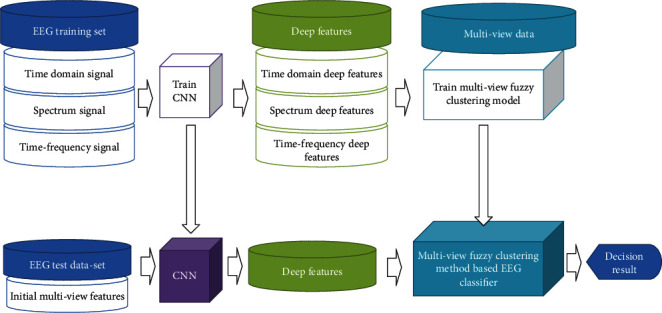
The proposed algorithm framework.

The algorithm framework is shown in Figure 4. First, epilepsy EEG data from multiple views is obtained. Fourier transform and wavelet transform were used to extract the spectrum signal, time domain signal, and time frequency signal of EEG data. Secondly, for each view signal, the classic DCNN is used for automatic feature extraction to reduce the sample dimension of each view. At this stage, the deep features of multiview epilepsy EEG data are obtained. Finally, a new classification method based on unsupervised multiview clustering results is used to obtain the final decision result.

### 4.2. Deep Feature Extraction

DCNN is used to extract the features of the data from various perspectives to obtain the deep spectrum features, deep time domain features, and deep time frequency features. Construct a multiview data set of epilepsy EEG based on deep features. The structure of the DCNN model used when extracting deep features from various perspectives is shown in Table 2.

### 4.3. Multiview Fuzzy Clustering Method-Based EEG Classifier

*X* = {*x*_1_, *x*_2_, ⋯, *x*_*K*_} is a multiview data set with *N* views. *X*^*i*^ represents the *i*-th view data, 1 ≤ *i* ≤ *N*. The *u*(1 ≤ *u* ≤ *K*) row vector represents the feature vector of the data object x_*u*_ in the *i*-th view space, where *n*_*i*_ is the characteristic dimension of the *i*-th perspective. The objective function of multiview FCM is as follows:

(1) $$\begin{array}{c} P\left(U,O,W\right)=\overset{N}{\underset{i=1}{\sum }}w_{i}\overset{C}{\underset{c=1}{\sum }}\overset{K}{\underset{\mu =1}{\sum }}\overset{n_{i}}{\underset{p=1}{\sum }}{\left(u_{c\mu }\right)}^{m}d\left(x_{\mu p}^{\left(i\right)},o_{cp}^{\left(i\right)}\right)+T_{w}\overset{N}{\underset{i=1}{\sum }}w_{i}\ln\left(w_{i}\right), \end{array}$$

(2) $$\begin{array}{c} \text{s}.\text{t}.\;\left\{\begin{array}{c} \overset{C}{\underset{c=1}{\sum }}u_{c\mu }=1,\;1\leq \mu \leq K,\\ \overset{N}{\underset{i=1}{\sum }}w_{i}=1. \end{array}\right. \end{array}$$

Parameter *m* is the fuzzy index, which is used to adjust the fuzzy degree of membership. *U* = (*u*_*cμ*_)_*C*×*N*_ is the membership matrix, where *u*_*cμ*_ represents the membership degree of the object *x*_*μ*_ belonging to the *c*-th cluster. *O*^(*k*)^ = {*o*_1_^(*k*)^, *o*_2_^(*k*)^, ⋯, *o*_*C*_^(*k*)^} is the vector of the *k*-th class center. *W* = (*w*_*i*_)_1×*N*_, where *w*_*i*_ represents the view *i* weights. *d*(*x*_*μp*_^(*i*)^, *o*_*cp*_^(*i*)^) represents the Euclidean distance between *x*_*μ*_ and *o*_*c*_^(*i*)^ in the *p*-dimensional space; *T*_*w*_ stands for regularization parameter. Construct the Lagrange function of Equation (2) as follows:

(3) $$\begin{array}{c} L\left(U,O,W\right)=\overset{N}{\underset{i=1}{\sum }}w_{i}\overset{C}{\underset{c=1}{\sum }}\overset{K}{\underset{\mu =1}{\sum }}\overset{\eta_{i}}{\underset{p=1}{\sum }}{\left(u_{c\mu }^{\left(i\right)}\right)}^{m}d\left(x_{\mu p}^{\left(i\right)},o_{cp}^{\left(i\right)}\right)+T_{w}\overset{N}{\underset{i=1}{\sum }}w_{i}\ln\left(w_{i}\right)+\overset{K}{\underset{\mu =1}{\sum }}\lambda_{\mu }\left(\overset{C}{\underset{c=1}{\sum }}u_{c\mu }^{\left(i\right)}-1\right)+\phi \left(\overset{N}{\underset{i=1}{\sum }}w_{i}-1\right). \end{array}$$

Both *λ*_*μ*_ and *ϕ* are Lagrange factors of Equation (2). When the minimum value of Equation (3) is taken, the following 5 necessary conditions must be met

(4) $$\begin{array}{c} \frac{\partial L}{\partial \lambda_{\mu }}=\overset{C}{\underset{c=1}{\sum }}u_{c\mu }^{\left(i\right)}-1=0, \end{array}$$

(5) $$\begin{array}{c} \frac{\partial L}{\partial u_{c\mu }^{\left(i\right)}}=\overset{N}{\underset{i=1}{\sum }}w_{i}\overset{n_{i}}{\underset{p=1}{\sum }}m{\left(u_{c\mu }^{\left(i\right)}\right)}^{m-1}d\left(x_{\mu p}^{\left(i\right)},o_{cp}^{\left(i\right)}\right)+\lambda_{\mu }=0, \end{array}$$

(6) $$\begin{array}{c} \frac{\partial L}{\partial o_{kp}^{\left(i\right)}}=2\overset{m}{\underset{\mu =1}{\sum }}{\left(u_{c\mu }^{\left(i\right)}\right)}^{m}\left(x_{\mu p}^{\left(i\right)}-o_{cp}^{\left(i\right)}\right)=0, \end{array}$$

(7) $$\begin{array}{c} \frac{\partial L}{\partial \phi }=\overset{N}{\underset{i=1}{\sum }}w_{i}-1=0, \end{array}$$

(8) $$\begin{array}{c} \frac{\partial L}{\partial w_{i}}=\overset{C}{\underset{c=1}{\sum }}\overset{K}{\underset{\mu =1}{\sum }}\overset{n_{i}}{\underset{p=1}{\sum }}{\left(u_{c\mu }^{\left(i\right)}\right)}^{m-1}d\left(x_{\mu p}^{\left(i\right)},o_{cp}^{\left(i\right)}\right)+T_{w}\left(\ln\left(w_{i}\right)+1\right)+\phi =0. \end{array}$$

By solving Equation (4)-(8), the necessary conditions that need to be met when Equation (1) obtains the minimum value under constraints are as follows:

(9) $$\begin{array}{c} o_{cp}^{\left(i\right)}=\frac{\sum_{\mu =1}^{K}{\left(u_{c\mu }^{\left(i\right)}\right)}^{m}x_{\mu p}^{\left(i\right)}}{\sum_{\mu =1}^{K}{\left(u_{c\mu }^{\left(i\right)}\right)}^{m}}, \end{array}$$

(10) $$\begin{array}{c} {u^{\left(i\right)}}_{c\mu }={\left(\overset{C}{\underset{e=1}{\sum }}{\left(\frac{\sum_{i=1}^{N}w_{i}\sum_{p=1}^{n_{i}}d\left(x_{\mu p}^{\left(i\right)},o_{cp}^{\left(i\right)}\right)}{\sum_{i=1}^{N}w_{i}\sum_{p=1}^{n_{i}}d\left(x_{\mu p}^{\left(i\right)},o_{ep}^{\left(i\right)}\right)}\right)}^{1/\left(m-1\right)}\right)}^{-1}, \end{array}$$

(11) $$\begin{array}{c} w_{i}=\frac{\exp\left(-D^{\left(l\right)}/T_{w}\right)}{\sum_{l=1}^{N}\text{x}\text{p}\left(-D^{\left(l\right)}/T_{w}\right)}, \end{array}$$

(12) $$\begin{array}{c} D^{\left(l\right)}=\overset{C}{\underset{c=1}{\sum }}\overset{K}{\underset{\mu =1}{\sum }}\overset{n_{l}}{\underset{p=1}{\sum }}{\left(u_{c\mu }^{\left(i\right)}\right)}^{m}d\left(x_{\mu p}^{\left(l\right)},o_{cp}^{\left(l\right)}\right). \end{array}$$

After the iterative optimization of the above parameters, we can use the following formula to complete the classification of EEG data. This is a new classification method based on the results of unsupervised multi-view clustering. The specific classification model is as follows:

(13) $$\begin{array}{c} f\left(x\right)=\max\left(W_{i}*U^{\left(i\right)}\left(x\right)\right), \end{array}$$

where *W*_*i*_ is obtained by Equation (11) and *U*^(*i*)^(*x*) is obtained by Equation (10).

The algorithm execution steps are as follows.

## 5. Experiment

### 5.1. Experiment Related Settings

In order to verify the effectiveness of the proposed algorithm, the experiment mainly conducts comparative analysis from two aspects. (1) When the proposed classification method based on unsupervised multiview clustering results is used to analyze whether deep features are used, the trend of classification performance changes. The general feature extraction methods used here are PCA [48] and LDA [49]. (2) In the case of deep features, the performance changes when using single-view classifiers and multiview classifiers. The comparison algorithms here mainly include FCM, SVM, RBF-NN, and multiview FCM. The indexes comparing the performance of epilepsy detection [50] are shown in Table 3. The larger the values of the two evaluation indicators shown in Table 3, the better the performance of the algorithm to be evaluated.

### 5.2. Experimental Data Description

The EEG database used in the experiment came from the Epilepsy Center of the University Hospital of Freiburg, Germany [51]. For 16 patients with refractory focal epilepsy, the epilepsy center recorded the patient's intracranial EEG during the preoperative epilepsy monitoring using invasive electrodes. The data acquisition process uses a Neuro NT digital video monitoring system that can record up to 128 channels of EEG signals. This system uses a 16-bit analog-to-digital converter with a sampling frequency of 256 Hz. Preprocess the collected raw data to obtain the EEG signal with 23 channels. The detailed statistics of the data used in the experiment are shown in Table 4.

### 5.3. Analysis of Experimental Results

**Figure 5:**
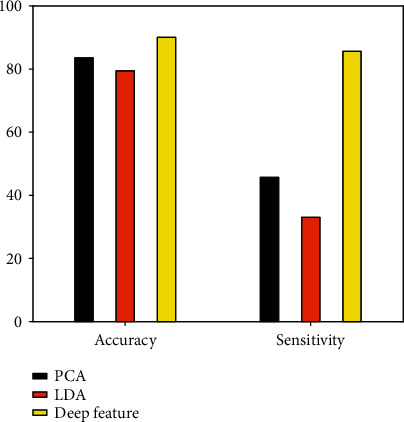
Schematic diagram of performance comparison under different feature extraction methods.

In order to analyze the influence of depth features on classification performance, a classifier is fixed, and the multiview FCM is selected here. The classifier is used to process feature data obtained by different feature extraction methods. The experimental data in Table 5 is the clustering performance obtained after processing the feature data extracted by different feature extraction methods using the multiview FCM algorithm. Intuitive feedback from the data in the table shows the depth features obtained based on DCNN feature extraction. After being processed by the multiview FCM classifier, the classification performance is significantly better than the other two traditional feature extraction methods. Comparing the two traditional feature extraction methods of PCA and LDA, the effect of PCA is better than that of LDA, but the effect is much worse than that of deep feature extraction.  Figure 5 intuitively shows the comparison of performance under different feature extraction methods.

**Figure 6:**
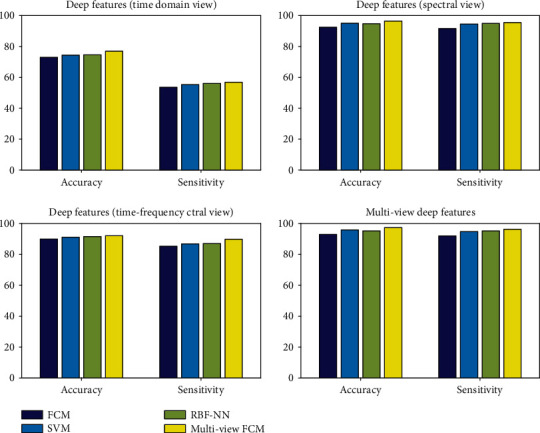
Performance comparison of various algorithms under various perspectives.

In order to analyze the classification performance of different classifiers on deep feature data, this paper selected three single-view classifiers and one multiview classifier. The different classifiers are applied to the deep feature data from different perspectives and the deep feature data from multiple perspectives for comparative analysis.  Table 6 compares the classification performance of different algorithms on the deep features of different perspectives. From the experimental data in the table, it can be observed that among the deep features under three vseparate perspectives, the classification performance on the time domain deep feature is the worst. Among them, the classification performance of the deep features under the spectrum perspective is the best. Regardless of the deep feature under which angle of view or the deep feature under multiple angles of view, the multiview FCM algorithm has the best classification performance. The classification performance under a single perspective is worse than that of multiview data, which further shows that multiview data is richer in information and more beneficial to classification processing.  Figure 6 gives a visual comparison of the performance of each algorithm under different perspectives.

## 6. Conclusion

This study proposes a new classification method based on unsupervised multiview clustering results. First, a multiview FCM clustering algorithm is introduced, and the training samples are used to train the center and weight of each view. Then the class center and weight of each view obtained by training are used to calculate the view weighted membership value of the new prediction sample. Finally, the classification label of the sample is obtained. The main contributions of the proposed method are as follows: (1) the deep feature can effectively reduce the data dimension while increasing the data separability; (2) the use of multiview data can improve the richness of data information, making decision results more stable and robust; and (3) the use of multiview classifiers improves the classification performance of traditional single-view classifiers. Experiments show that the proposed method is more automated, intelligent, and efficient than traditional EEG detection based on machine learning.

## Figures and Tables

**Algorithm 1:**
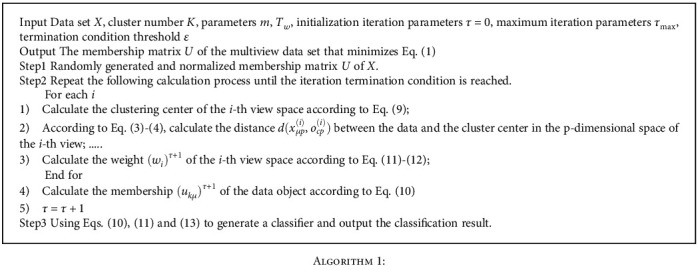
Algorithm 1:

**Table 1:** Common EEG data feature.

Name	Details
Time domain feature	The EEG signal is a time series signal that changes with time. The discrete points in the signal represent the energy intensity at a certain moment or the voltage value measured at that moment. In this study, the original EEG signal is directly used as the feature data from the time domain perspective.
Spectrum feature	The EEG signal is mainly divided into 6 frequency intervals, namely, *δ*_1_ (0-2 Hz), *δ*_2_ (2-4 Hz), *θ* (4-8 Hz), *α* (8-15 Hz), *β* (15-30 Hz), and *λ* (30-60 Hz). The spectral features of seizures are mainly distributed between 4 Hz and 30 Hz. For the initial EEG signal, the feature data under the spectral view were obtained by Fourier transform.
Time-frequency feature	Wavelet packet decomposition (WPD) is performed on the time-domain signal to obtain feature data from a time-frequency view. The sampling interval of the large frequency domain is set at 2 Hz, and the decomposition level of the wavelet transform is 6 layers. The time-frequency feature selection interval is 4 Hz-30 Hz.

**Table 2:** Network structure of deep feature extraction from various views.

Parameter/feature	Deep time domain feature	Deep spectrum feature	Deep time-frequency feature
Layer 1 (input layer)	Original matrix: 23∗256 Convolution kernel: 1∗128 Step size: 1	Original matrix: 23∗27 Convolution kernel: 4∗4 Step size: 1	Original matrix: 256∗23∗14 Convolution kernel: 129∗1∗1 Step size: 1
Layer 2 (convolutional layer)	Feature map: 1@23∗129 Convolution kernel: 1∗65 Step size: 30	Feature map: 20@20∗24 Convolution kernel: 8∗8 Step size: 1	Feature map: 1@128∗23∗24 Convolution kernel: 65∗4∗4 Step size: 1
Layer 3 (convolutional layer)	Feature map: 30@23∗6 Convolution kernel: 4∗33 Step size: 1	Feature map: 10@13∗17 Convolution kernel: 8∗8 Step size: 1	Feature map: 30@64∗24∗11 Convolution kernel: 30∗4∗4 Step size: 1
Layer 4 (convolutional layer)	Feature map: 20@20∗33 Convolution kernel: 8∗18 Step size: 1	—	Feature map: 20@32∗17∗8 Convolution kernel: 17∗8∗1 Step size: 1
Layer 5 (convolutional layer)	Feature map: 10@13∗16	—	Feature map: 10@16∗10∗8
Layer 6 (fully connected layer)	The 10 feature maps of size 13∗16 output from the convolutional layer of the fifth layer are converted into a vector of size 1∗2080, which is used as the input of the fully connected layer. Feature map: 1@1∗102	The 10 feature maps of size 13∗17 output by the convolutional layer of the third layer are converted into a vector of size 1∗2210, which is used as the input of the fully connected layer. Feature map: 1@1∗512	The 10 feature maps of size 16∗10∗8 output by the convolutional layer of the fifth layer are converted into a vector of size 1∗12800, which is used as the input of the fully connected layer. Feature map: 1@1∗2048
Layer 7 (fully connected layer)	Feature map: 1@1∗100	Feature map: 1@1∗100	Feature map: 1@1∗1024
Layer 8 (fully connected layer)	Output: 1∗1024 vector	Output: 1∗512 vector	Feature map: 1@1∗100
Layer 9 (fully connected layer)	—	—	Output: 1∗2048 vector

**Table 3:** Explanation of evaluation index.

Index	Index calculation formula	Remarks
Accuracy	TN + TP/TP + TN + FP + FN	TP is the number of samples that correctly predict the seizure fragments. FN is the number of samples that predict epileptic seizure fragments as nonepileptic seizures. FP is the number of samples that predict nonseizure fragments as seizures. TN is the number of samples that correctly predict the non-seizure segment.
Sensitivity	TP/TP + FN	

**Table 4:** Experimental data.

Number	Signal length during seizures (seconds)	Signal length during the absence of epilepsy (seconds)
1	389	40287
2	202	69882
3	387	132382
4	549	129898
5	364	384984
6	186	220384
7	284	134443
8	583	153433
9	860	130239
10	798	71023
11	453	135668
12	237	174994
13	468	117894
14	346	256768
15	276	67974
16	189	86432

**Table 5:** Performance comparison of different feature extraction methods under multiview FCM classifier.

Feature extraction method\index	Accuracy	Sensitivity
PCA	83.22	45.68
LDA	79.43	33.18
Deep feature	89.75	85.52

**Table 6:** Performance comparison of different algorithms on different depth features.

Feature	Classifier	Accuracy	Sensitivity
Deep features (time domain view)	FCM	73.02	53.57
	SVM	74.38	55.36
	RBF-NN	74.62	56.11
	Multiview FCM	76.98	56.87
Deep features (spectral view)	FCM	92.45	91.56
	SVM	95.03	94.48
	RBF-NN	94.74	94.96
	Multiview FCM	96.42	95.52
Deep features (time-frequency view)	FCM	89.89	85.30
	SVM	91.10	86.85
	RBF-NN	91.54	87.08
	Multiview FCM	92.12	89.83
Multiview deep features	FCM	92.98	92.02
	SVM	95.86	94.78
	RBF-NN	95.25	95.21
	Multiview FCM	**97.38**	**96.26**

## Data Availability

The labeled data set used to support the findings of this study is available from the corresponding author or upon request.
